# Dental and oral health assessments in the German National Cohort (NAKO)

**DOI:** 10.1186/s12903-025-05454-z

**Published:** 2025-01-28

**Authors:** Stefanie Samietz, Katrin Borof, Katrin Hertrampf, Ghazal Aarabi, Antonio Ciardo, Hannah Finke, Daniel Hagenfeld, Jan Kühnisch, Maurice Rütters, Sebastian-Edgar Baumeister, Stefan Lars Reckelkamm, Ti-Sun Kim, Thomas Kocher, Wolfgang Ahrens, Hermann Brenner, Carina Emmel, Beate Fischer, Amand Führer, Karin Halina Greiser, Jasmin Grischke, Kathrin Günther, Volker Harth, Stefanie Jaskulski, André Karch, Thomas Keil, Yvonne Kemmling, Alexander Kluttig, Lilian Krist, Oliver Kuss, Michael Leitzmann, Claudia Meinke-Franze, Karin B. Michels, Nadia Obi, Anette Peters, Nicole Pischon, Tobias Pischon, Sabine Schipf, Börge Schmidt, Henning Teismann, Stefan Rupf, Birte Holtfreter

**Affiliations:** 1 Department of Prosthetic Dentistry, Gerodontology and Biomaterials, University Medicine Greifswald, Greifswald, Germany; 2 Department of Periodontics, Preventive and Restorative Dentistry, Centre for Dental and Oral Medicine, University Medical Centre Hamburg-Eppendorf, Hamburg, Germany; 3 Department of Oral and Maxillofacial Surgery, University Hospital Schleswig-Holstein, Kiel, Germany; 4 Section of Periodontology, Department of Conservative Dentistry, Clinic for Oral, Dental and Maxillofacial Diseases, Heidelberg Faculty of Medicine, Heidelberg University, Heidelberg, Germany; 5 Department of Orthodontics, University Hospital, Tübingen, Germany; 6 Clinic for Periodontology and Conservative Dentistry, University of Münster, Münster, Germany; 7 Department of Conservative Dentistry and Periodontology, Ludwig-Maximilian University Hospital, Ludwig-Maximilian University Munich, Munich, Germany; 8 Institute of Health Services Research in Dentistry, University of Münster, Münster, Germany; 9 Department of Restorative Dentistry, Periodontology and Endodontology, University Medicine Greifswald, Greifswald, Germany; 10 Leibniz Institute for Prevention Research and Epidemiology – BIPS, Bremen, Germany; 11 Division of Clinical Epidemiology and Aging Research, German Cancer Research Center (DKFZ), Heidelberg, Germany; 12 Institute for Medical Informatics, Biometry and Epidemiology, University of Duisburg-Essen, Essen, Germany; 13 Institute for Epidemiology and Preventive Medicine, University of Regensburg, Regensburg, Germany; 14 Institute for Medical Epidemiology, Biometrics and Informatics, Martin Luther University Halle-Wittenberg, Halle (Saale), Germany; 15 Division of Cancer Epidemiology, German Cancer Research Centre (DKFZ), Heidelberg, Germany; 16 Department of Prosthetic Dentistry and Biomedical Materials Science, Hannover Medical School, Hannover, Germany; 17 Institute for Occupational Medicine and Maritime Medicine (ZfAM), University Medical Centre Hamburg-Eppendorf, Hamburg, Germany; 18 Institute for Prevention and Cancer Epidemiology, Faculty of Medicine and Medical Centre, University of Freiburg, Freiburg, Germany; 19 Institute of Epidemiology and Social Medicine, University of Münster, Münster, Germany; 20 Institute of Social Medicine, Epidemiology and Health Economics, Charité - Universitästmedizin Berlin, Berlin, Germany; 21 Institute of Clinical Epidemiology and Biometry, University of Würzburg, Würzburg, Germany; 22 State Institute of Health I, Bavarian Health and Food Safety Authority, Erlangen, Germany; 23 Department of Epidemiology, Helmholtz-Centre for Infection Research (HZI), Braunschweig, Germany; 24 Institute for Biometrics and Epidemiology, German Diabetes Center, Leibniz Center for Diabetes Research at Heinrich Heine University Düsseldorf, Düsseldorf, Germany; 25 Institute for Community Medicine, University Medicine Greifswald, Greifswald, Germany; 26 Institute of Epidemiology, Helmholtz Zentrum München, German Research Center for Environmental Health (GmbH), Neuherberg, Germany; 27 Institute for Medical Information Processing, Biometry, and Epidemiology (IBE), Faculty of Medicine, Ludwig-Maximilians-Universität, Munich, Germany; 28 Molecular Epidemiology Research Group, Max-Delbrueck-Centre for Molecular Medicine in the Helmholtz Association (MDC), Berlin, Germany; 29 Charité - Universitätsmedizin Berlin, corporate member of Freie Universität Berlin and Humboldt-Universität zu Berlin, Berlin, Germany; 30 Zahn- und Prophylaxe Centre Priv.-Doz. Dr. Nicole Pischon, Berlin Schönefeld, Germany; 31 Biobank Technology Platform, Max-Delbrueck-Centre for Molecular Medicine in the Helmholtz Association (MDC), Berlin, Germany; 32 Synoptic Dentistry, Saarland University, Homburg/Saar, Germany

**Keywords:** Epidemiology, Cohort study, Cross-sectional, Caries, Periodontitis, Dental status, Temporomandibular disorders, Oral health

## Abstract

**Background:**

Despite considerable improvements in oral health in recent decades, caries and periodontitis are still widespread, ranking among the most prevalent diseases worldwide and requiring future research. The German National Cohort (NAKO Gesundheitsstudie, NAKO) is a large-scaled, multidisciplinary, nationwide, multi-centre, population-based, prospective cohort study with oral examinations that aims to provide a resource to study risk factors for major diseases. The aim of the present article is to provide the methodological background, to report on the data quality, and to present initial results of the oral examinations.

**Methods:**

During baseline examinations (2014–2019), a total of 205,184 persons aged 19–74 years has been examined in 18 study centres, including, among others, a dental interview, stimulated saliva sampling, and recording of the numbers of present teeth and prostheses (standard Level 1 program). As part of the Level 2 program that was offered to 20% randomly selected participants, each study centre selected one of three modules, one of them being the Level 2 oral examination. This extended program was carried out in a subgroup of 20,828 participants, including collection of detailed information on the dental and prosthetic status as well as on periodontal, cariological and functional aspects. To ensure reliability and reproducibility, study nurses were trained and calibrated by dental experts. In addition, a reliability study was conducted among 794 Level 1 and 359 Level 2 participants, reporting intra class correlation and kappa coefficients.

**Results:**

Intra class correlation and kappa coefficients for observer agreement and reliability were consistently above 0.7, indicating good to excellent reliability of all dental measurements. For example, intra class correlation was 0.937 for the number of present teeth (Level 1), 0.740 for mean probing depth (PD) and 0.797 for active mouth opening. An initial inspection of the data showed that the median number of present teeth was 27, of which on average 6.9 teeth were healthy and caries-free. Average mean PD was 1.92 mm. An orthodontic treatment was reported by 35.5% of participants.

**Discussion:**

Overall, the dental study protocol was feasible and successfully integrated into the NAKO’s overall assessment program. However, rigorous support of the study centres by dental professionals was required to ensure high quality data. In summary, high-quality data collection within the NAKO pave the way for future investigation of potential risk factors for oral diseases and links between oral and systemic diseases and conditions.

**Supplementary Information:**

The online version contains supplementary material available at 10.1186/s12903-025-05454-z.

## Background

Recent decades have been characterized by global public health impacts such as increasing social inequalities, ageing populations, a global pandemic, and an increased prevalence of noncommunicable diseases [1]. Resultingly, important determinants of health, such as access to public health service, common risk factor profiles, living conditions and environmental exposures, are changing over time. Therefore, large-scale cohort studies have the potential to detect such changes and use these insights to improve prevention.

Oral health is a ‘fundamental component of health and physical and mental wellbeing’ [2]. It is multi-faceted and ‘includes the ability to speak, smile, smell, taste, touch, chew, swallow and convey a range of emotions through facial expressions with confidence and without pain, discomfort and disease of the craniofacial complex’ [2]. The two most prevalent oral diseases, dental caries and periodontitis, are a major public health problem worldwide [3, 4] and the main causes of tooth loss across the lifespan [5]. It is thus of great importance to identify prevention and risk factors in order to improve primary prevention or treatment measures. Comprehensive data from large cohort studies on the most common oral diseases are therefore urgently needed.

Untreated caries in permanent teeth is the most prevalent health condition worldwide, affecting 2.3 billion people in 2017 [6]. Only few studies have been published to assess caries trends in adults worldwide [7–18]. In Germany, the number of decayed or filled teeth per person and the number of persons affected by caries decreased consistently over the last two decades [9, 16, 17, 19]. Fluoridated toothpastes and salt fluoridation have been central to reducing dental caries in Germany over the past five decades [20–24], with toothpaste adoption in the 1970s driving early declines both in adolescents and adults in West Germany and post-reunification use contributing to rapid decreases in East Germany [17, 25]. The introduction of salt fluoridation in 1991 [26] provided complementary, population-wide benefits, especially in underserved groups or regions with inconsistent toothpaste use, helping to sustain and extend caries reductions into the twenty-first century. In 2005, fluoridated table salt had a market share of around 67% in Germany [27]. While both interventions have significantly reduced caries prevalence, limited studies have directly compared their independent and combined effects, leaving gaps in understanding their relative contributions.

Chronic periodontitis is a highly prevalent oral disorder [28–31], as evidenced by a recent meta-analysis of periodontitis prevalence data from studies performed between 2011 and 2020, which estimated the prevalence to be approximately 62%, with severe forms observed in 23.6% of cases. Based on data from the Global Burden of Disease Study, severe periodontitis affected 1067 million people in 2021 [28]. Periodontitis is caused by a destructive inflammatory host response after the microbiome of the crevice has shifted towards pathogenesis composition [32], and leads to a loss of connective tissue and supporting tissue and, if left untreated, tooth loss. While inconsistent information about recent trends of periodontitis prevalence was reported [33–36], treatment needs are expected to increase further due to higher numbers of exposed natural teeth, a shift to more aged populations and global population growth [28, 37]. In 2014, about 51.6% and 64.6% of 35–44- and 65–74-year-old Germans, respectively, had moderate or severe periodontitis [37], which illustrates the high need for treatment in Germany [38].

Periodontitis does not only affect the oral cavity, but is potentially linked to several systemic diseases. Specifically, evidence suggests that chronic periodontitis affects metabolic control in diabetes patients [39, 40], and may also be linked to cardiovascular diseases [41–43]. Both diseases have a high and increasing prevalence, incidence, and morbidity, and cardiovascular diseases are responsible for most deaths worldwide [44]. Therefore, periodontitis should be understood as a disease with a considerable impact on general health.

Tooth loss is the endpoint of the most prevalent dental diseases, dental caries and periodontitis [5], and leads to reduced masticatory function [45] and to substantial decline in oral health-related quality of life (OHRQoL) [46, 47]. In recent decades, the prevalence of edentulism (toothlessness) has declined, while the number of retained teeth has increased, especially in older subjects [37]. Globally, age-standardized prevalence of edentulism was 3.3% .

Malocclusion and temporomandibular disorders are among the less common oral diseases. The prevalence of malocclusion and temporomandibular disorders in adults is unclear. According to a recent systematic review, 25% of European children and adolescents had a Class II malocclusion, while 5% had an anterior crossbite [48]. Studies among German children showed an increased (> 4 mm) and negative overjet in 16–31% and 1–3%, respectively [49–51]. An increased (> 4 mm) or negative overbite was found in 21–46% and 3–17% of German children, respectively [49, 51, 52]. Temporomandibular disorders are one of the most common orofacial pain conditions [53] with varying prevalence across surveys. While 2.7% of the population in north-east Germany was affected by temporomandibular joint pain and muscle disorders around the year 2000 [54], in Sweden it was 10% of the adult population around 2011 [55]. Temporomandibular disorders can impair the quality of life to large extent [56, 57].

The NAKO was designed to investigate possible causes and mechanisms for the onset and progression of frequent chronic diseases [58]. The objective of the present paper is to describe the rationales and design of the oral examinations in the NAKO study. Furthermore, we refer to details about the recording of the dental interview, the dental and prosthetic status, coronal caries, periodontitis, temporomandibular disorders, and orthodontic measures. We describe the standardized data collection, the quality assurance measures undertaken, the data management, and we assess observer agreement and reliability using a reliability study. To provide an insight into the data, preliminary results for selected oral variables are presented.

## Methods/design

### Study design

The NAKO is a multidisciplinary, nationwide, multi-centre, population-based cohort study that aims to investigate the development and aetiology of diseases, identify risk factors and enhance early detection and prevention of diseases. To develop a notion of the required sample size, the following aspects had to be considered : i) enable the statistical analysis of a great variety of potential research questions, ii) balance the study size against the depth of phenotyping and detail of data collected, iii) allow the development of multivariable risk models, iv) for rarer forms of disease or pre-clinical phenotypes ‘make a meaningful contribution to international cohort consortia’ .

A comprehensive description of the study population, its recruitment and the baseline examination and data collection of the NAKO study was published elsewhere [1]. Briefly, the study aimed to recruit 200,000 participants aged 20 to 69 years from the general population living in the adjacent areas of 18 study centres based on samples drawn randomly from municipal registries. A sex and age stratified sampling design was used, with equal balance among sexes, and 10.0% of participants in each 10-year age group from 20–39, and 26.7% in each 10-year age group from 40 to 69. The baseline study program included a standardized, computer-assisted face-to-face interview, biomedical examinations, self-report questionnaires, data from various imaging techniques (for example 3D ultrasound of the heart), collection of biosamples, and a whole-body MRI (30,000 participants). By design, data collection comprised two levels of intensity. The standard Level 1 program was offered to all participants. In addition, in-depth examinations were offered to 20% randomly selected participants (Level 2 program). Each study centre selected one of three modules (1 of 3 exam system) to be carried out in all Level 2 participants, while the other two modules were only performed in a subgroup of 100 participants. Some study centres chose 2 out of 3 modules and carried out each module on one-half of all Level 2 participants.

Between March 2014 and September 2019, a total of 205,184 persons aged 20–74 years were recruited and examined at 18 study centres (covering rural and urban regions) across Germany (Level 1 examinations), while 57,051 participants passed through Level 2 examinations. Differences to the number of participants reported in Peters et al. [1] can be explained by withdrawn consent after data transfer.

Oral examinations were included in both Level 1 (Supplemental Fig. 1A, C) and Level 2 examinations (Supplemental Fig. 1B, D; as part of the 1 of 3 exam system; Level 2 oral examinations were performed in eight centres and a reduced number of Level 2 oral examinations was performed in the remaining 10 centres). Examination protocols were developed to ensure a maximum compatibility with study protocols from other German studies (data pooling), namely the German Oral Health Studies (Deutsche Mundgesundheitsstudien) [59] and the Studies of Health in Pomerania [60].

### Level 1 dental interview and oral examinations

A dental interview (*N* = 189,158) was carried out by means of a touchscreen device using a combination of self-completion questions, including also the German Oral Health Impact Profile (OHIP-G5) questionnaire [61] in order to assess the OHRQoL (Table 1). The OHIP-G5 reflects the individual’s perception of oral health and its impact on life on a multidimensional level considering biopsychosocial aspects related to oral health [62, 63].

**Table 1:** Overview on Level 1 dental questionnaire and examinations

Recordings/Question	Levels/possible answers
*Dental questionnaire*	
Has a dentist ever diagnosed you with periodontitis or periodontosis, i.e. inflammation of the periodontium?	Yes/ no/ don’t know
Do you have a dental implant? (dental implants are screwed into the jaw)	Yes, upper jaw/ yes, lower jaw/ yes, upper and lower jaw/ no/ don’t know
Do you have loose teeth??	Yes/ no
Do your gums bleed when brushing your teeth?	Yes/ no
*OHIP-G5: For each Oral Health Impact Profile (OHIP) question, participants were asked how frequently they had experienced the problem in the last month*	
Have you had painful aching in your mouth?	Never/ hardly ever/ occasionally/ fairly often/ very often
Have you felt uncomfortable about the appearance of your teeth, mouth dentures or jaws?	Never/ hardly ever/ occasionally/ fairly often/ very often
Have you felt that there has been less flavour in your food because of problems with your teeth, mouth, dentures or jaws?	Never/ hardly ever/ occasionally/ fairly often/ very often
Have you had difficulty doing your usual jobs because of problems with your teeth, mouth, dentures or jaws?	Never/ hardly ever/ occasionally/ fairly often/ very often
Have you had difficulty chewing any foods because of problems with your teeth, mouth, dentures or jaw?	Never/ hardly ever/ occasionally/ fairly often/ very often
*Dental examinations*	
Tooth count	Number of present teeth, including third molars
Number of removable dentures	No prosthesis/ one prosthesis/ two prostheses

Level 1 oral examinations were conducted in all participants (*N* = 189,205) and included the counting of present teeth, including third molars (range 0–32 teeth). This included healthy teeth, teeth with carious lesions and teeth with fillings or crowns, but no dental implants. Furthermore, the number of removable full or partial dentures was documented. The reasons for non-feasibility were described (Table 1).

### Level 2 oral examinations

In Level 2 (Table 2), conducted among a subgroup of participants (*N* = 20,943), the tooth status (including caries status), prosthetic status, periodontal status, orthodontic and functional status, and temporomandibular disorders were recorded. The dentures were removed from the mouth before the oral examination. The participants were asked whether and, if so, at which position dental implants were located. The following examinations were carried out according to the halfmouth design. The respective jaw half (left or right side) was randomly assigned. All permanent teeth, including third molars, were examined. The Level 2 dental status was recorded using the ParoStatus software (ParoStatus.de GmbH, Berlin, Germany), with study-specific adjustments according to the Level 2 recording protocol. The recordings were registered web-based. A printed copy was also maintained as a backup.

**Table 2:** Overview on Level 2 dental examinations

Examination part	Dental examination/ Questions	Recordings	Examination level
Prosthetic status	Dental status	Tooth extracted or not present/ tooth present/ implant/ decayed tooth/ dental status examination refused or not feasible	Tooth
	Prosthetic status	Healthy or no prosthetic replacement or no restauration/ filling/ inlay, partial crown, crown or veneer/ prosthodontic attachments (e.g. double crown, crown with attachment for a removable denture, anchor)/ pontic/ tooth replaced by removable partial dentures/ examination refused or not feasible	Tooth
Prostheses	Prostheses in lower and upper jaw	no prosthetic reconstruction/ prosthesis retained by wrought-wire-claps or provisional prosthesis/ prosthesis retained by cast claps/ prosthesis retained by telescopic crowns or precision attachments (telescopic/ conus/ double crown/ any kind of precision attachment)/ removable complete denture/ not assessable	Jaw
Periodontal status	Heart disease record card	Question: Do you have a heart disease record card? Possible answers: No/ yes/ don’t know	Subject
	Periodontal pocket depths	Periodontal pocket depth in mm Examination refused/ Examination not feasible	Site; halfmouth; mesiobuccal and midbuccal sites
Orthodontic and functional status	Orthodontic treatment	Question: Do or did you have any kind of orthodontic treatment with braces? Possible answers: No (no treatment)/ at present/ before the age of 18/ after the age of 18/ don’t know/ answer refused	Subject
	Pain	Question: Did you have pain in the face, in the jaw, or in front or behind your ears within the last months? Possible answers: Yes/ no/ don’t know/ refused to answer	Subject
	Dislocation	Question: Have you ever had a dislocation of your lower jaw, so that you were not able to close your mouth? Possible answers: no/ yes/ don’t know	Subject
	Jaw mobility	Active mouth opening in mm/ Examination refused/ Examination not feasible	Subject
	Jaw mobility	Maximum active mouth opening in mm Examination refused/ Examination not feasible	Subject
	Pain assessment	Pain during maximum active mouth opening assessments Joint pain – no/ yes, one the right side/ yes, on the left side/ yes, on both sides/ examination not feasible/ examination refused Muscle pain—no/yes, one the right side/ yes, on the left side/ yes, on both sides/ examination not feasible/ examination refused	Subject
	Overjet	Overjet in mm/ Examination refused/ Examination not feasible	Subject
	Overbite	Overbite in mm/ Examination refused/ Examination not feasible	Subject
	Muscle palpation	Palpation of Musculus masseter and Musculus temporalis on left and right side using a pressure algometer No pain/ mild pain/ moderate pain/ severe pain/ examination refused/ examination not feasible	Subject

#### Dental status

The tooth status was recorded independently of the tooth restoration and classified as follows: tooth extracted or not present; tooth present (a tooth was registered as healthy if there were no carious cavitated lesions or restorations; structural defects, cervical defects, abrasion, attrition and erosion of the teeth were not recorded; teeth with fissure sealings were also coded as healthy); implant; decayed tooth (decayed teeth were registered with primary or secondary caries at the cavitation level according to WHO criteria by visual inspection [64], confirmed by pressureless penetration of the rounded periodontal probe; root residuals were also documented in this category; non-cavitated carious lesions were assessed as healthy and were not recorded); examination was refused by participants or was not recordable.

#### Prosthetic status

The prosthetic status was recorded as follows: healthy or no prosthetic replacement or no restoration; fillings (all filled teeth were recorded here. Recording was performed independently of the filling material, e.g. amalgam, composite or cement, and independent of the tooth surface-specific extent or size; if there was a carious lesion at the edges of the filling or crown, this finding was registered separately in the "dental status" section); inlay, partial crown, crown or veneer (registration was carried out independently of the materials used, e.g. gold, ceramic, metal-ceramic, etc., and regardless of the extent and size); prosthodontic attachments, e.g. double crown, crown with attachment for a removable denture, anchor, etc. (a simple wire clasp or model casting clasp was not registered here); pontic (if a tooth was lost and replaced by a fixed partial denture pontic); tooth replaced by a removable denture (denture sections without an artificial tooth were registered as missing); examination refused or not feasible (examination was refused by the participant or examination of the teeth was not possible, for example, due to limited mouth opening). Possible combinations of dental and prosthetic status are listed in Table 3.

**Table 3:** Possible combinations of dental and prosthetic status

Dental status	Prosthetic status
Tooth extracted or not present	Healthy or no prosthetic replacement or no restoration
	Pontic
	Tooth replaced by removable denture
Tooth present	Healthy or no prosthetic replacement or no restoration
	Fillings
	Inlay, partial crown, crown, veneer
	Double crown, crown with attachment for a removable denture, anchor, etc
Implant	Healthy or no prosthetic replacement or no restoration
	Crown
	Double crown, crown with attachment for a removable denture, anchor, etc
	Tooth replaced by removable denture
Decayed tooth	Healthy or no prosthetic replacement or no restoration
	Fillings
	Inlay, partial crown, crown, veneer
	Double crown, crown with attachment for a removable denture, anchor, etc

Additionally, the presence of removable prosthetic dentures in the upper and lower jaw was documented separately as no prosthetic reconstruction, prosthesis retained by wrought-wire-clasps or provisional prosthesis, prosthesis retained by cast claps, prosthesis retained by telescopic crowns or precision attachments, removable complete denture, or not assessable (as a single category).

#### Periodontal status

Participants with a heart disease record card (for needed endocarditis prophylaxis) and participants who were not sure whether they had a heart disease record card were not periodontally examined. Probing depths (PD) were recorded at two sites (mesiobuccal and midbuccal) according to the half-mouth method (same side as dental status) including third molars. A 1 mm-scaled periodontal probe was used (PCP-UNC 15, Hu-Friedy, Chicago, IL, USA) with a force of approximately 0.25 N. PD represents the distance between the free gingival margin and the pocket base. Measurements were down rounded to the nearest millimetre and recorded in full millimetres.

#### Functional and orthodontic status

First, the participant was asked about any previous or current orthodontic treatment with braces (and the time of treatment) and about possible pain in the facial area (Table 2). Then, the functional status for jaw mobility or mouth opening was examined in all participants, including edentates. Participants with a known tendency to dislocate the temporomandibular joint were excluded from jaw mobility examinations, which included the active mouth opening and the maximum active mouth opening. Participants were asked to open their mouth as much as possible on their own without provoking pain. The examination was not recorded if the participant was edentulous in the anterior region and did not wear prostheses. While the participants keep their mouth open, the distance between the upper and lower incisal edges of the central incisors was documented in full millimetres. Afterwards, the maximum active mouth opening was measured. The participant was asked to open the mouth as wide as possible, even if he was in pain. Again, the distance between the upper and lower incisal edges of the central incisors was documented as described above. The participant was then asked whether and where (left or right side) they felt muscle or joint pain at maximum active mouth opening. Overjet and overbite were measured with a ruler in full millimetres.

Finally, bilateral muscle palpation of the masseter and temporalis muscles was performed using a pressure algometer (Wagner Force Ten FDX Compact, Wagner Instruments, Greenwich, CT, USA) with a standardized force. The application force of the pressure algometer was increased within 1–2 s to the intended strength of 4.5Newton. After each measurement, the participant was asked about possible pain (recorded as no, mild, moderate or severe pain). The variable ‘palpation’ was coded as yes if mild, moderate or severe pain was registered for at least one of the four positions palpated with the pressure algometer (Musculus masseter and Musculus temporalis on the left and right side); otherwise coded as ‘no’.

#### Saliva sampling

A stimulated saliva sample was taken from all participants using a paraffin gum (1 g paraffin, GC Germany GmbH, Bad Homburg vor der Höhe, Germany). The last meal and/or drink should have been at least 30 min ago. The participants were asked to rinse the oral cavity at least 10 min with 0.2 ml of tap water before the saliva sampling. The participants were motivated to actively chew the paraffin to stimulate salivation. During one minute, the saliva was continuously spat into a collection cup. When the amount of saliva after one minute was less than 0.9 ml, the participant was asked to chew the paraffin chewing gum for a further minute and saliva was collected. For storage, the collected saliva was aliquoted into two cryotubes with a capacity of 500 μl each. The samples were immediately coded and temporarily stored in dry ice. Until the end of the day, cryotubes were frozen at −80 °C [58].

### Quality assurance measures

Numerous measures were implemented to assure the high-quality standards for oral examinations [65], including intensive theoretical and practical training of all study nurses by an experienced dentist (J.K.). Qualified study nurses had to fulfil a number of specific conditions: i) successful participation at a theoretical and a practical training course, ii) correct implementation of the specific dental standard operating procedure (SOP), iii) successful certification and, if applicable, re-certification (S.S.; S.R.) at regular intervals, and iv) completion of required site visits. A dental advisor (a dentist) was appointed for each Level 2 centre to act as a contact person and dental trainer. All certified trainers were familiar with the SOP, had completed a certification workshop and had participated in regular working group meetings every two years. The detailed SOPs with appendices and hand books are available on request.

### Data processing

Personal data were processed according to the concept on data privacy protection and IT development for the NAKO [1, 66]. The Independent Trust Centre curates all IDs and stored informed consents. The dental data quality control and plausibility checks have been processed by the dental working group. Data were directly integrated in two data integration centres, one at the University Medicine Greifswald for the northern study centres and one at the German Cancer Research Centre (DKFZ) for the southern study centres in Germany [58, 65, 67].

### Data plausibility and completeness

After completion of the data collection, the data was subsequently checked and extensively corrected as part of quality assurance. The corrected data is made available for all subsequent data usage applications. First, the data was corrected according to the comments entered by the examiners during the dental examination (including, for example, input corrections). Second, to identify implausible values, various plausibility checks were performed: implausible combinations (for example, a high number of present teeth and the presence of 1 or more prostheses; Level 1) were identified for future applications for data use and recommendations for data processing were given. For orthodontic measurements, plausibility limits were defined and measurements outside limit ranges were set to missing (overbite: [−20; 20]; overjet [−50; 50]; (maximum) active mouth opening [0; 100]. Detailed quality assurance reports are available on request.

### Reliability study

We performed a reliability study, in which 6,000 NAKO participants were re-examined after on average 270 days (*N* = 794; standard deviation (SD) 82.7; range 35–434) and 274 days (*N* = 359; SD 80.5; range 49–434) for Level 1 and Level 2 examinations, respectively. We calculated intra class correlation coefficients (ICCs; using single and average measures, as appropriate), percentage agreement, and kappa coefficients for Level 1 and Level 2 examinations restricting measurement pairs to those done by the same examiner at both time points (minimum number of observations per group set to 30).

### Statistical methods

For Level 2 participants maximum PD and mean PD were calculated, considering all available site measurements (28 at maximum). The Decayed Teeth (DT) index, the Filled Teeth (FT) index, the Decayed Filled Teeth (DFT) index, and the Decayed Missing Filled Teeth (DMFT) index were determined (half-mouth, excluding third molars; range 0–14).

For continuous data, means ± standard deviations and medians (25% and 75% quantiles) were presented. Numbers (percentages) were shown for categorical data.

Marginal means and locally estimated scatterplot smoothing (LOESS) plots were assessed for Level 1 tooth counts and Level 2 mean PD, the DFT Index, and the active mouth opening distance. The dataquier package (version 2.0.1) was used in R 4.3.2 to compute marginal plots (using the acc_margins function) and LOESS plots (using the acc_loess function) [68]. For marginal plots, adjusted marginal means were calculated using equally weighted marginal effects of the factor-variable ‘study centre’. For each level of ‘study centre’, marginal means were calculated including 95% confidence intervals. Marginal distributions were plotted together with box plots combined with violin plots, or with count plots. Also, the overall mean and the overall deviation from the mean (± 1 standard deviation) are displayed. In case of oddities the marginal mean is displayed in red.

LOESS was conducted to examine the impact of ‘study centre’ on the measurements over time [69]. If a fitted curve exceeds the confidence band of dashed red lines of the overall distribution a severe shift is observed, indicating persistent trends. In case of persistent trends in selected levels of ‘study centre’, systematic changes in measurements over time would be implied.

From the reliability study, repeatedly measured data of both Level 1 and Level 2 dental status were used to assess observer agreement and reliability. Value pairs for (maximum) active mouth opening were restricted to those with an absolute difference of less than 20 to avoid excessive differences between repeated measurements due to entry errors. Likewise, value pairs for overjet and overbite were restricted to those with an absolute difference of less than 10. For continuous measurements intra class correlation coefficients (ICCs, with 95% confidence intervals; CI) were assessed. For categorical variables, agreement and kappa (with standard errors; SE) were calculated. For palpation variables, only % agreement was reported because kappas could not be determined as palpation variables were scored zero in most data pairs (> 98%).

## Results

### Study participants

**Fig. 1:**
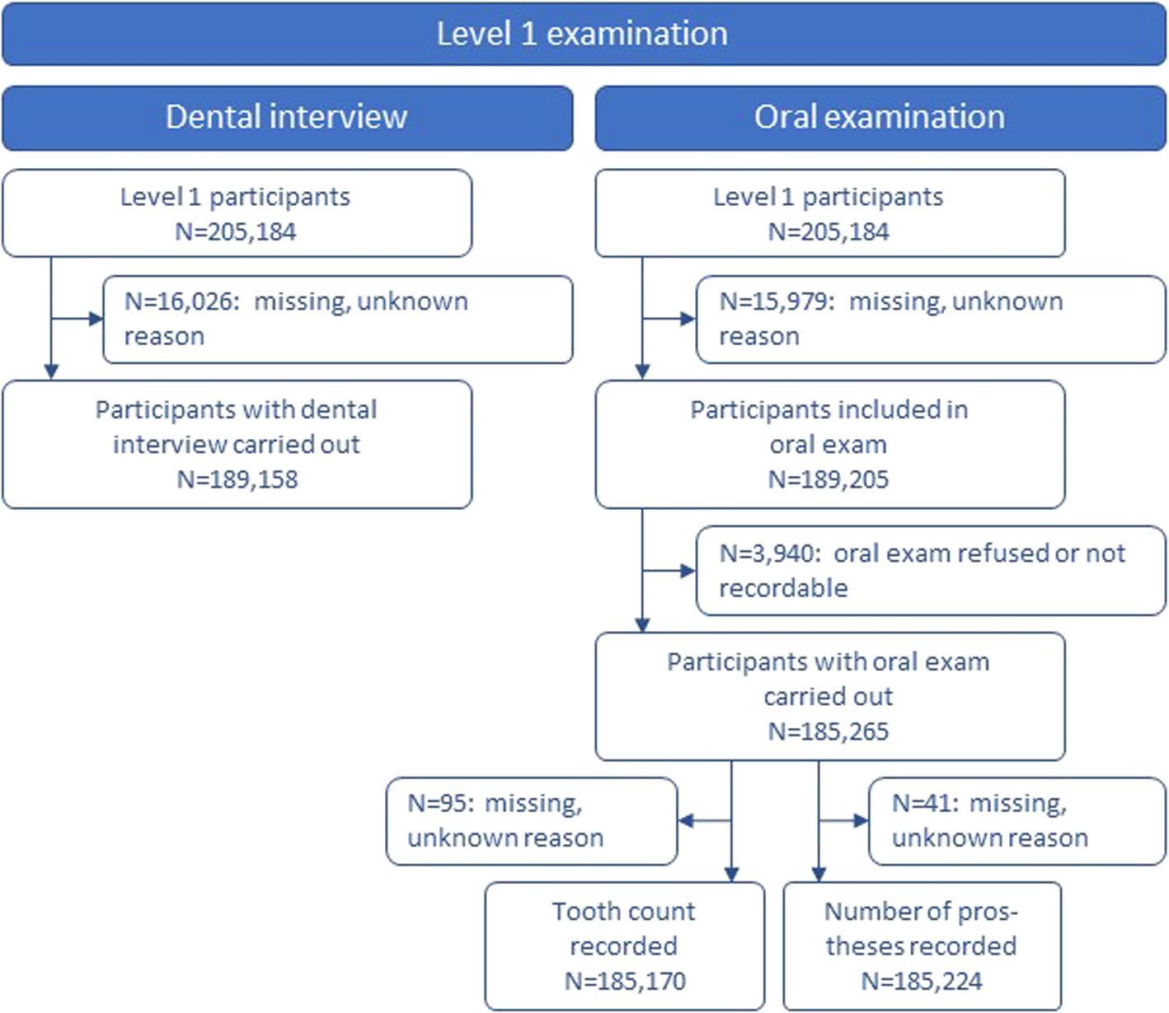
Flowchart for the Level 1 dental interview and oral examination data

**Fig. 2:**
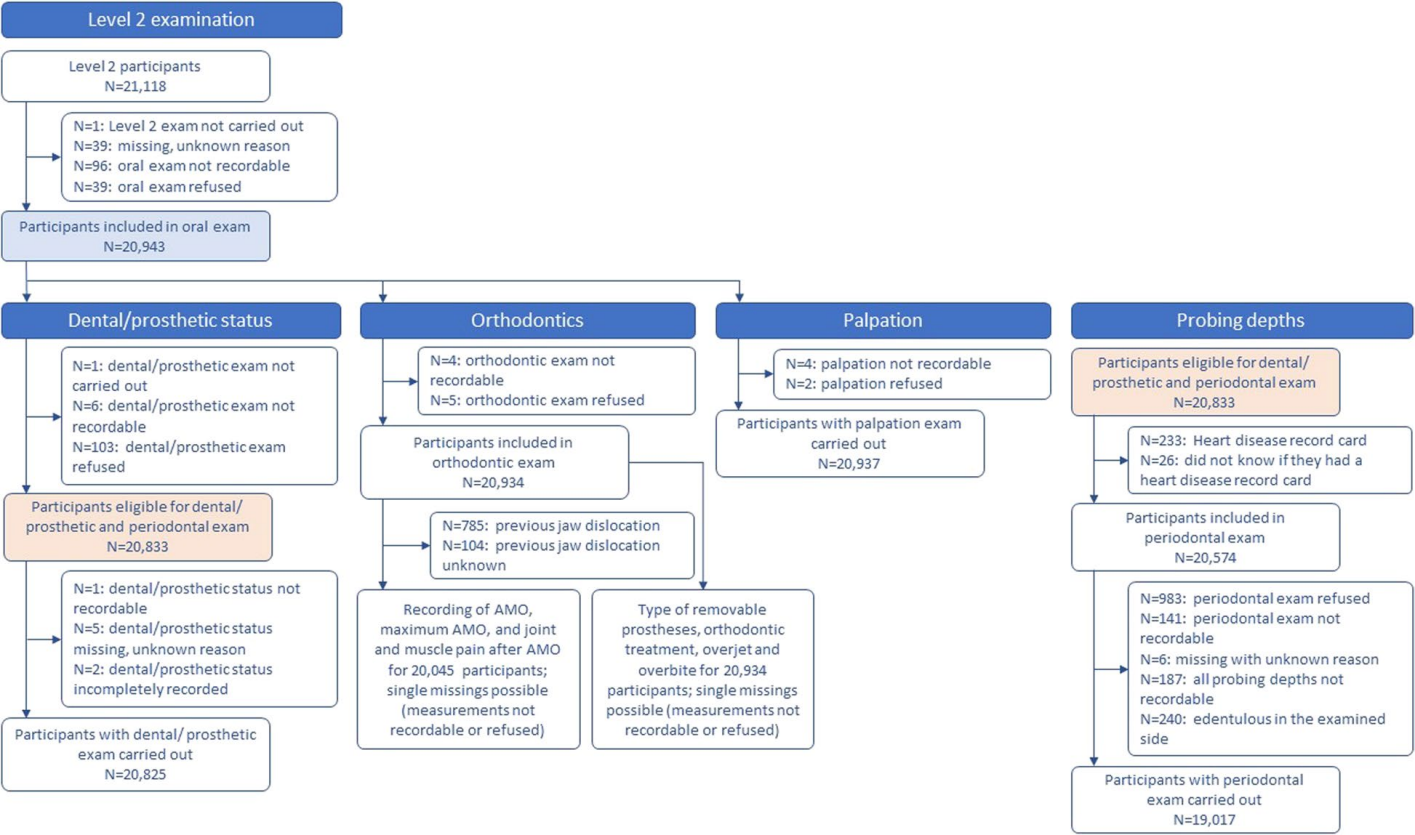
Flowchart for the Level 2 examination (dental/prosthetic status, orthodontics, palpation, and probing depths)

The age distribution of the total population, including the 205,184 participants, was as follows: 19,983 people (9.7%) aged 19–29 years, 21,684 (10.6%) aged 30–39 years, 53,232 (25.9%) aged 40–49 years, 55,013 (26.8%) aged 50–59 years and 55,272 (26.9%) aged 60–75 years. Flow charts for the derivation of study participants for Level 1 examinations (interview and oral examination) and Level 2 examinations (dental/prosthetic status, probing depths, orthodontics, palpation) are shown in Figs. 1 and 2.

### Marginal means and monitoring of measurements over time by study centre

**Fig. 3:**
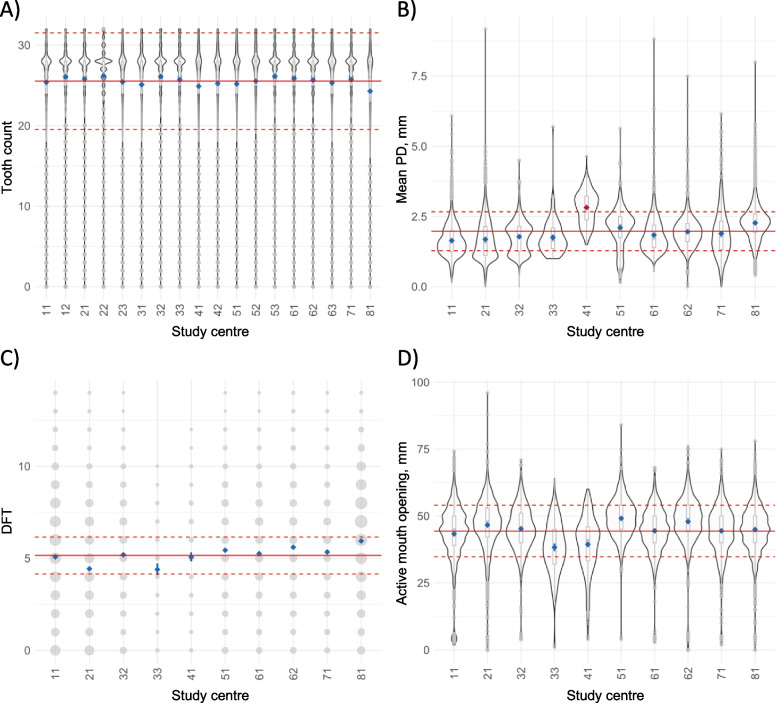
Study centre margins (blue) for **A**) full-mouth tooth counts (including third molars; Level 1), **B**) mean probing depth (PD), **C**) the Decayed-Filled-Teeth (DFT) Score and **D**) the active mouth opening distance (Level 2) after adjustment for age and sex using statistical models. Marginal distributions (using box plots combined with violin plots, or count plots) are additionally shown. Also, the overall mean (red solid line) and the deviation from the mean (± 1 standard deviation; red dashed lines) are displayed. In case of oddities the marginal mean is displayed in red

**Fig. 4:**
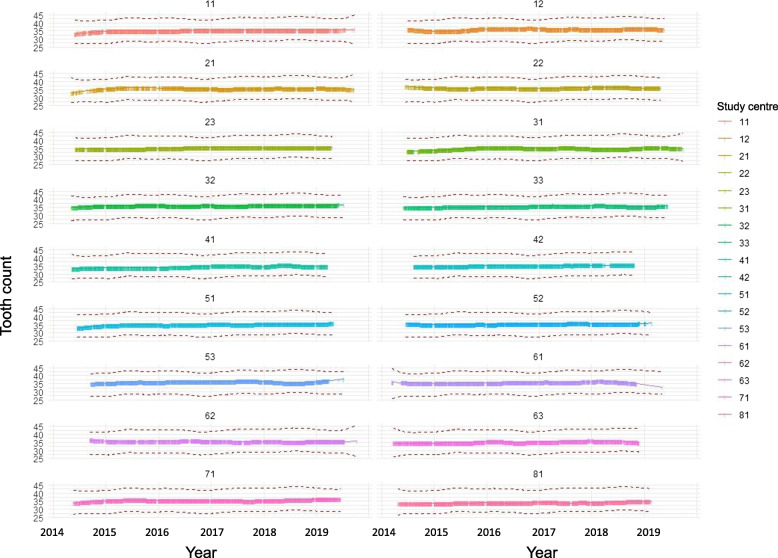
LOESS-smoothed curves for each study centre for full-mouth tooth counts (including third molars; Level 1) after adjustment for age and sex using statistical models

**Fig. 5:**
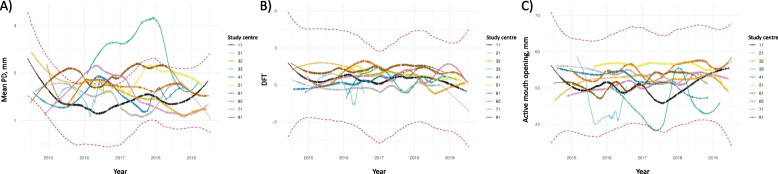
LOESS-smoothed curves for each study centre for **A**) mean probing depth (PD), **B**) the Decayed-Filled-Teeth (DFT) Score and **C**) the active mouth opening distance (Level 2) after adjustment for age and sex using statistical models. The red dashed lines represent the confidence interval of a LOESS curve for the whole data

Marginal means plots identified no systemic outliers across study centres for tooth counts, the DFT index and the active mouth opening distance (Fig. 3). For mean PD, the marginal mean for one study centre was higher compared to all other study centres and above confidence limits. LOESS plots (Figs. 4 and 5) did not identify systematic trends over time within study centres. Furthermore, each of the study centre specific LOESS curves were not separated from LOESS curves of the other study centres.

### Reliability using repeated measures from the reliability study

#### Level 1 oral examination

Results from the reliability study are presented in Table 4. For the number of prostheses, kappa for repeated measurement was 0.92 (SE 0.03). The ICC for the number of teeth including third molars was 0.94 (95% CI: 0.93, 0.95).

**Table 4:** Results from the reliability study: intra class correlation (ICCs) and kappa coefficients for Level 1 and Level 2 examinations restricting measurement pairs to those done by the same examiner at both time points

	Level of analysis	N obs	Measure for ICCs	ICC (95% CI)	Agreement, %	Kappa (SE)
Level 1 examinations						
Number of prostheses (0/1/2)	Subject	783	-	-	98.7%	0.92 (0.03)
Number of teeth, incl. third molars	Subject	794	Single	0.94 (0.93, 0.95)	-	-
Level 2 examinations						
Dental status	Tooth	4,956	-	-	97.4%	0.88 (0.01)
Prosthetic status	Tooth	4,956	-	-	100.0%	1.00 (0.01)
Probing depth, mm	Site	7,731	Average	0.74 (0.73, 0.75)		
Prostheses in upper jaw	Subject	359	-	-	96.1%	0.75 (0.03)
Prostheses in lower jaw	Subject	359	-	-	97.8%	0.78 (0.04)
Active mouth opening, mm	Subject	318	Single	0.80 (0.75, 0.83)	-	-
Maximum active mouth opening, mm	Subject	321	Single	0.91 (0.89, 0.93)	-	-
Overjet, mm	Subject	333	Single	0.88 (0.85, 0.90)	-	-
Overbite, mm	Subject	339	Single	0.79 (0.74, 0.82)	-	-
Palpation of the right temporalis muscle	Subject	359	-	-	97.8%	ND
Palpation of the right masseter muscle	Subject	359	-	-	96.4%	ND
Palpation of the left temporalis muscle	Subject	359	-	-	98.9%	ND
Palpation of the left masseter muscle	Subject	359	-	-	96.7%	ND

For palpation variables, only % agreement was reported because kappas could not be determined (ND, not determined)

*Abbreviations*: *ICC* intra class correlation coefficient, *N obs.* number of observations, *CI* confidence interval, *SE* standard error

#### Level 2 oral examination

For the dental and the prosthetic status (tooth level data) intra class kappa was 0.88 (SE 0.01) and 1.00 (SE 0.01), respectively. For probing depths (site level) the intra class ICC (average rater) was 0.74 (95% CI: 0.73, 0.75). For assessments of the type of removable prostheses in the upper and lower jaw, intra class kappas were 0.75 and 0.78, respectively. For assessments of active mouth opening, maximum active mouth opening, overjet and overbite, intra class ICCs were 0.80 (95% CI: 0.75, 0.83), 0.91 (95% CI: 0.89, 0.93), 0.88 (95% CI: 0.85, 0.90), and 0.79 (95% CI: 0.74, 0.82), respectively. For palpation variables, intra class percentages of agreement ranged between 96.4 and 98.9%.

### Distribution of oral health related variables

#### Level 1 dental interview

Dental interview data were recorded in 205,184 participants at the time of data transfer. The proportion of missing values varied across dental interview items (around 8%). Thirty percent reported a diagnosis of periodontitis, 5.9% reported to have loose teeth, and 16.7% reported to have bleeding gums (Table 5). Dental implants, either in the lower and/or the upper jaw, were reported in 14.4% of participants. According to OHIP questions (counting very often, often or occasionally; referring to problems with the teeth, mouth, dentures or jaw within that last months), 1.9% reported painful aching, 10.1% felt uncomfortable about the appearance, 2.4% reported less flavour in their food, 1.8% reported difficulties doing their usual jobs, and 8.6% reported difficulties in chewing.

**Table 5:** Results of the responses to the dental interview questions (*N* = 205,184)

Item	Diagnosis of periodontitis or periodontosis	Loose teeth	Bleeding gums	Dental implants	
Answers				Answers	
Yes	61,537 (30.0%)	12,163 (5.9%)	34,329 (16.7%)	No	156,834 (76.4%)
No	98,073 (47.8%)	173,130 (84.4%)	154,626 (75.4%)	Yes, in upper jaw	12,270 (6.0%)
Don’t know	29,438 (14.3%)	1 (0.0%)	0 (0%)	Yes, in lower jaw	10,389 (5.1%)
				Yes, in upper and lower jaw	6,771 (3.3%)
				Don’t know	2,753 (1.3%)
Missings				Missings	
Not specified	94 (0.1%)	99 (0.1%)	96 (0.1%)	Not specified	93 (0.1%)
Module missing	16 (0.01)	3,765 (1.8%)	107 (0.1%)	Module missing	48 (0.02%)
Item missing	16,026 (7.8%)	16,026 (7.8%)	16,026 (7.8%)	Item missing	16,026 (7.8%)
Item	OHIP-G5: Painful aching	OHIP-G5: Uncomfortable about appearance	OHIP-G5: Less flavour in your food	OHIP-G5: Difficulty doing your usual jobs	OHIP-G5: Difficulty chewing
Answers					
Very often	1,235 (0.6%)	2,074 (1.0%)	532 (0.3%)	426 (0.2%)	1,543 (0.8%)
Fairly often	2,655 (1.3%)	3,834 (1.9%)	958 (0.5%)	691 (0.3%)	2,981 (1.4%)
Occasionally	20,411 (9.9%)	14,790 (7.2%)	3,332 (1.6%)	2,591 (1.3%)	13,123 (6.4%)
Hardly ever	44,918 (21.9%)	26,014 (12.7%)	9,822 (4.8%)	7,436 (3.6%)	20,164 (9.8%)
Never	119,647 (58.3%)	142,139 (69.3%)	174,190 (84.9%)	177,676 (86.6%)	151,005 (73.6%)
Don’t know	1 (0.0%)	1 (0.0%)	1 (0.0%)	2 (0.0%)	3 (0.0%)
Missings					
Not specified	96 (0.1%)	92 (0.04%)	91 (0.04%)	93 (0.1%)	93 (0.1%)
Module missing	194 (0.1%)	213 (0.1%)	231 (0.1%)	242 (0.1%)	245 (0.1%)
Item missing	16,027 (7.8%)	16,027 (7.8%)	16,027 (7.8%)	16,027 (7.8%)	16,027 (7.8%)

*Abbreviations*: *OHIP-G5* Oral Health Impact Profile German 5 (including 5 questions)

#### Level 1 oral examination

The median number of present teeth was 27 (25% quantile: 25; 75% quantile: 28; Table 6). A removable denture in one jaw was documented in 9,965 (5.4%) and in both jaws in 10,244 (5.5%) participants.

**Table 6:** Descriptive results for level 1 and level 2 dental examinations at baseline of the German National Cohort (NAKO)

		Descriptive data	
	N	Mean ± SD Median (Q25%; Q75%)	N (%)
**Level 1 examinations (fullmouth)**			
Number of present teeth (including third molars)	185,170	25.5 ± 6.0 27 (25; 28)	
Number of prostheses	185,224		
0			165,015 (89.1%)
1			9,965 (5.4%)
2			10,244 (5.5%)
**Level 2 dental/prosthetic status (halfmouth)**			
Examined quadrants	20,833		
Left			7,892 (37.9%)
Right			12,941 (62.1%)
Dental status (tooth level)	333,745		
Tooth extracted or not present			63,711 (19.1%)
Tooth present			264,913 (79.5%)
Implant			2,633 (0.8%)
Decayed tooth			1,488 (0.4%)
Missing			503 (0.2%)
Number of teeth	20,825	12.3 ± 2.7 13 (12; 14)	
Healthy caries-free teeth (excl. third molars)	20,825	6.9 ± 3.6 7 (4; 9)	
DT Index (excl. third molars)	20,825	0.1 ± 0.4 0 (0; 0)	
FT Index (excl. third molars)	20,825	5.3 ± 3.0 5 (3; 7)	
DFT Index (excl. third molars)	20,825	5.4 ± 3.0 6 (3; 8)	
MT Index (excl. third molars)	20,825	1.7 ± 2.7 1 (0;2)	
DMFT Index (excl. third molars)	20,825	7.1 ± 3.6 7 (5; 10)	
**Level 2 Probing depths (halfmouth)**			
Probing depth, mm (site level)	490,346	1.9 ± 1.0 2 (1; 2)	
Mean probing depth, mm (subject level)	19,017	1.91 ± 0.71 1.88 (1.39; 2.33)	
Maximum probing depth, mm (subject level)	19,017	3.29 ± 1.45 3 (2; 4)	
**Level 2 Orthodontics**			
Type of removable prosthetic restoration in lower jaw	20,934		
No prosthetic reconstruction			19,194 (91.7%)
Prosthesis retained by wrought-wire-claps or provisional prothesis			272 (1.3%)
Prosthesis retained by cast claps			528 (2.5%)
Prosthesis retained by telescopic crowns or precision attachments			584 (2.8%)
Removable complete denture			210 (1.0%)
Not assessable			146 (0.7%)
Type of removable prosthetic restoration in upper jaw	20,934		
No prosthetic reconstruction			19,533 (93.3%)
Prosthesis retained by wrought-wire-claps or provisional prothesis			234 (1.1%)
Prosthesis retained by cast claps			437 (2.1%)
Prosthesis retained by telescopic crowns or precision attachments			512 (2.5%)
Removable complete denture			107 (0.5%)
Not assessable			111 (0.5%)
Orthodontic treatment	20,665		
No			13,349 (64.5%)
Now			60 (0.3%)
Yes, before the age of 18			6,495 (31.4%)
Yes, after the age of 18			409 (2%)
Yes, before the age of 18 and now			11 (0.05%)
Yes, after the age of 18 and now			6 (0.03%)
Yes, before and after the age of 18			283 (1.4%)
Yes, before and after the age of 18 and now			11 (0.05%)
Don’t know			41 (0.02%)
Overbite, mm	20,182	3.4 ± 2.02 3 (2; 5)	
Overjet, mm	20,104	3.1 ± 3.1 3 (2; 4)	
Active mouth opening, mm	19,808	45.2 ± 10.1 46 (40; 51)	
Maximum active mouth opening, mm	19,734	50.7 ± 8.4 51 (46; 56)	
**Level 2 Palpation**			
Palpation of the right Musculus temporalis	20,937		
No pain			20,584 (98.3%)
Mild pain			116 (0.55%)
Moderate pain			20 (0.1%)
Severe pain			13 (0.1%)
Not assessable			156 (0.75%)
Answer refused			48 (0.2%)
Palpation of the right Musculus masseter	20,937		
No pain			20,456 (97.7%)
Mild pain			215 (1.0%)
Moderate pain			56 (0.3%)
Severe pain			10 (0.05%)
Not assessable			153 (0.7%)
Answer refused			47 (0.2%)
Palpation of the left Musculus temporalis	20,936		
No pain			20,590 (98.4%)
Mild pain			108 (0.5%)
Moderate pain			32 (0.1%)
Severe pain			8 (0.04%)
Not assessable			152 (0.7%)
Answer refused			46 (0.2%)
Missing			1 (0.0%)
Palpation of the left Musculus masseter	20,937		
No pain			20,456 (97.7%)
Mild pain			214 (1.0%)
Moderate pain			56 (0.3%)
Severe pain			11 (0.05%)
Not assessable			155 (0.7%)
Answer refused			45 (0.2%)
Palpation over all four positions	20,735		
No			20,163 (97.2%)
yes (mild, moderate or severe pain for at least one position)			572 (2.8%)

*Abbreviations*: *N* number, *%* percentage, *Q25%* 25% quantile, *Q75%* 75% quantile, *SD* standard deviation

#### Level 2 oral examination

Oral examinations were performed halfmouth (Table 6): 37.9% on the left and 62.1% on the right side (due to technical problems at the beginning). The dental status was recorded in 20,825 participants. On tooth level, 63,711 teeth (19.1%) were found to be extracted or not present; at 2,633 (0.8%) of all tooth positions implants were registered. Caries was registered for 1,488 teeth (0.4%). At the subject level, an average of 6.9 teeth (SD 3.6) were healthy and caries-free, 0.1 teeth (SD 0.4) were decayed, 5.3 teeth (SD 3.0) were filled and 1.7 teeth (SD 2.7) were missing. The average DMFT Index was 7.1 (SD 3.6).

PD values ranged between 0 and 15 mm. Mean PD was 1.92 mm on average (SD 0.70). Maximum PD was 3.35 mm on average (SD 1.49). A removable denture in the lower and upper jaw was registered in 7.7% and 6.2%, respectively. An orthodontic treatment was reported by 35.5% of participants. The average overbite was 3.4 mm (SD 2.2) and the average overjet was 3.1 mm (SD 3.1). The average active mouth opening distance was 45.2 mm (SD 10.1).

## Discussion

This report describes the study protocol, the standardized recording of the oral examination, and some preliminary results from the NAKO, a multicentre, population-based epidemiologic cohort study of 205,184 women and men, covering a wide age range (19–74 years). The results of the data quality assessments and the plausibility of the collected data demonstrated that the oral examination protocol was well implemented. Data quality was assessed by observer agreement and reliability measures derived from a reliability study and close data monitoring, as in other epidemiologic studies with oral examination components, such as the German Oral Health Studies [70] or the Studies of Health in Pomerania [60]. Furthermore, by ensuring a maximum compatibility of the study protocol with other European studies (data pooling), the NAKO offers an approach for linking the study with other studies, for example the German Oral Health Studies (Deutsche Mundgesundheitsstudien), the Studies of Health in Pomerania, or the Hamburg City Health Study [71].

Both in Level 1 and Level 2 participants, comprehensive oral examination data were recorded by regularly trained, calibrated, and certified study nurses using standardized protocols, requiring extensive coordination at the expert level. The enormous quality assurance effort was well reflected in i) good to excellent reliability of dental measurements and ii) non-presence of systemic effects of examiner and centre on oral measurements. First, using repeatedly collected data from the reliability study, good to excellent reliability was shown (excluding examiners with less than 30 observations; Table 4). It is important to note that as baseline and reliability study examinations were on average 270 days apart, reliability measures in this study reflect both, changes in the dental and/or periodontal status (be it natural changes or changes due to therapies) as well as intra-rater measurement error. Thus, we assume that our reliability estimates are conservative and that the true reliability is higher (i.e., measurement error is lower). However, it should be noted that although the dental examiners were trained and process calibrated by professional dentists before and during data collection, validity was not assessed against a gold standard. Second, measurements were monitored over time and by study centre (Figs. 3, 4 and 5). Marginal means plots did not show systematic deviations for any of the dental variables. Several conclusions can be deducted from LOESS plots (Figs. 4 and 5). Firstly, no systematic trends over time were identified. Secondly, no persistent trends in selected levels of ‘study centre’ were detected, implying no systematic changes in measurements over time. Thirdly, as complete separation of the LOESS curve for one study centre compared to all other study centres was not apparent, systematic differences in measurements, which are independent of time, were unlikely. Taken together, the quality measures implemented ensured the high quality of the data.

The standardized protocols for conducting Level 1 and Level 2 oral examinations served as essential tools to achieve harmonization across study sites. In conjunction with training courses and web seminars conducted by certified dentists, the manual played a key role in enabling study nurses without formal dental training to systematically collect pertinent dental observations. This holistic approach facilitated the effective implementation of a multifaceted interdisciplinary oral examination program within the NAKO by competent non-dental personnel. As a result, the assessment of dental, periodontal, and oral health status was uniformly standardized, along with cariologic, orthodontic, and functional assessments.

The comprehensive oral examination facilitates the assessment of dental and oral health in different age groups of the German population. By scheduling a follow-up examination of participants, incidence estimates can be derived for periodontitis, dental caries, and newly placed prosthetic restorations. This capability is particularly unique due to the prospective and longitudinal nature of the cohort design, a feature currently observed in only a limited number of large-scaled studies. The comprehensive and interdisciplinary nature of the investigations allows dental findings to be contextualized within the broader medical landscape. This is particularly interesting for parameters such as number of teeth and oral hygiene status, both of which can serve as indicators of individual health practices. Furthermore, the combination of Level 1 and Level 2 dental data with information from comprehensive computerized interviews and various medical examinations, including data from various imaging techniques (for example, magnetic resonance imaging) and biospecimens, allows for the evaluation of potential associations between oral diseases, specifically periodontitis, and various systemic conditions and diseases using advanced measurement techniques. Also, assessment of the functional status allows for collaborative interdisciplinary analyses with orthopaedic specialists, for example. Taken together, this results in a wide range of analysis options.

One important methodological consideration should be noted when interpreting the Level 2 data. Given the impracticality and cost constraints associated with the comprehensive analysis of all teeth, assessments of tooth status, caries presence, restoration status, and probing depth were performed on either the left or right side of the mouth. This approach is justified by the assumption of a symmetric intraoral distribution of caries and probing depths [72–74], thus preserving the validity of the results. However, it is important to recognize that partial diagnosis may lead to underestimation of periodontal prevalence and severity [75, 76]. In addition, the use of risk models may attenuate effect estimates, potentially biasing associations toward the null [77]. It is also pertinent to note that due to a software documentation error, the randomized selection of the oral hemisphere did not begin until May 2016, with only the right side being used prior to that. However, given the expectation of minimal asymmetry at the level of the jaw [72–74], the validity of the results remains unaffected by this procedural change.

The collection of dental data in the NAKO provides a solid foundation for dental epidemiologic research. The collaboration of different dental health research centres, the nationwide recruitment of survey participants, and the use of established standardized examination protocols facilitate comparisons with large-scale international cohort studies. Thus, there is a strong potential to analyse associations of oral diseases and various medical conditions and diseases in order to develop new strategies for prevention, early detection and risk stratification.

## Supplementary Information

Supplementary Material 1.

## Data Availability

Data of the NAKO are generally not available to the public due to strict data protection regulations. However, scientists can apply for data use according to the official usage regulation specifications. Please refer to https://transfer.nako.de for further information.

## Abbreviations

AMO: Active Mouth Opening
CI: Confidence Interval
DT: Decayed Teeth
DFT: Decayed Filled Teeth
DKFZ: German Cancer Research Centre
DMFT: Decayed Missing Filled Teeth
DMS: Deutsche Mundgesundheitsstudien
FT: Filled Teeth
ICC: Intra-rater Correlation Coefficients
LOESS: Locally estimated scatterplot smoothing
NAKO: German National Cohort, NAKO Gesundheitsstudie
OHIP-G5: German Oral Health Impact Profile
OHRQoL: Oral Health-Related Quality of Life
PD: Probing depth
SD: Standard deviation
SE: Standard error
SOP: Standard Operating Procedure
