# Supplementary material for: Dental and oral health assessments in the German National Cohort (NAKO)
Source: BMC Oral Health. 2025 Jan 28;25:149. doi: 10.1186/s12903-025-05454-z (PMC11773730; doi:10.1186/s12903-025-05454-z)
Supplement: Supplementary file 1 — Supplementary Material 1. [file 12903_2025_5454_MOESM1_ESM.docx]

# **Online Supplemental Material**

# Dental and oral health assessments in the German National Cohort (NAKO)

Stefanie Samietz*, Katrin Borof*, Katrin Hertrampf, Ghazal Aarabi, Antonio Ciardo, Hannah Finke, Daniel Hagenfeld, Jan Kühnisch, Maurice Rütters, Sebastian Edgar Baumeister, Stefan Lars Reckelkamm, Ti-Sun Kim, Thomas Kocher, Wolfgang Ahrens, Hermann Brenner, Carina Emmel, Beate Fischer, Amand Führer, Halina Greiser, Jasmin Grischke, Kathrin Günther, Volker Harth, Stefanie Jaskulski, André Karch, Thomas Keil, Yvonne Kemmling, Alexander Kluttig, Lilian Krist, Oliver Kuss, Michael Leitzmann, Claudia Meinke-Franze, Karin B. Michels, Nadia Obi, Anette Peters, Nicole Pischon, Tobias Pischon, Sabine Schipf, Börge Schmidt, Henning Teismann, Stefan Rupf*, Birte Holtfreter*

* contributed equally


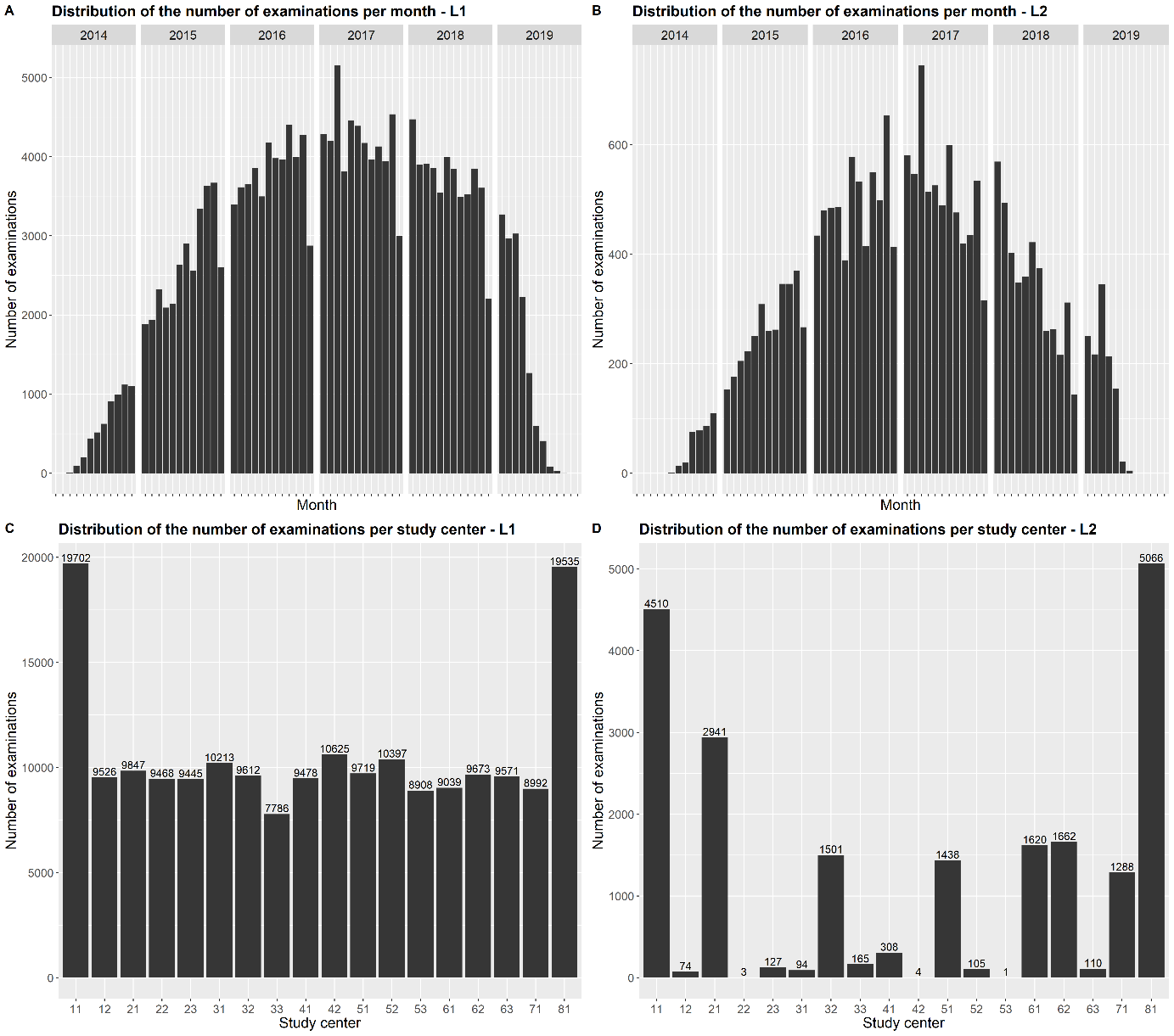


Supplemental Figure 1. Number of Level 1 (L1; left) and Level 2 (L2; right) dental examinations by year/month (A-B) and study centre (C-D).
